# Diversity in warning coloration: selective paradox or the norm?

**DOI:** 10.1111/brv.12460

**Published:** 2018-08-27

**Authors:** Emmanuelle S. Briolat, Emily R. Burdfield‐Steel, Sarah C. Paul, Katja H. Rönkä, Brett M. Seymoure, Theodore Stankowich, Adam M. M. Stuckert

**Affiliations:** ^1^ Centre for Ecology & Conservation, College of Life & Environmental Sciences University of Exeter Penryn Campus, Penryn, Cornwall, TR10 9FE U.K.; ^2^ Centre of Excellence in Biological Interactions, Department of Biological and Environmental Science University of Jyväskylä Jyväskylä, 40014 Finland; ^3^ Department of Chemical Ecology Bielefeld University Universitätsstraße 25, 33615, Bielefeld Germany; ^4^ Organismal and Evolutionary Biology Research Programme, Faculty of Biological and Environmental Sciences University of Helsinki Helsinki, 00014 Finland; ^5^ Department of Biology Colorado State University Fort Collins CO 80525 U.S.A.; ^6^ Department of Fish, Wildlife, and Conservation Biology Colorado State University Fort Collins CO 80525 U.S.A.; ^7^ Department of Biological Sciences California State University Long Beach CA 90840 U.S.A.; ^8^ Department of Biology East Carolina University 1000 E Fifth St, Greenville NC 27858 U.S.A.

**Keywords:** aposematism, continuous variation, polymorphism, polytypism

## Abstract

Aposematic theory has historically predicted that predators should select for warning signals to converge on a single form, as a result of frequency‐dependent learning. However, widespread variation in warning signals is observed across closely related species, populations and, most problematically for evolutionary biologists, among individuals in the same population. Recent research has yielded an increased awareness of this diversity, challenging the paradigm of signal monomorphy in aposematic animals. Here we provide a comprehensive synthesis of these disparate lines of investigation, identifying within them three broad classes of explanation for variation in aposematic warning signals: genetic mechanisms, differences among predators and predator behaviour, and alternative selection pressures upon the signal. The mechanisms producing warning coloration are also important. Detailed studies of the genetic basis of warning signals in some species, most notably *Heliconius* butterflies, are beginning to shed light on the genetic architecture facilitating or limiting key processes such as the evolution and maintenance of polymorphisms, hybridisation, and speciation. Work on predator behaviour is changing our perception of the predator community as a single homogenous selective agent, emphasising the dynamic nature of predator–prey interactions. Predator variability in a range of factors (e.g. perceptual abilities, tolerance to chemical defences, and individual motivation), suggests that the role of predators is more complicated than previously appreciated. With complex selection regimes at work, polytypisms and polymorphisms may even occur in Müllerian mimicry systems. Meanwhile, phenotypes are often multifunctional, and thus subject to additional biotic and abiotic selection pressures. Some of these selective pressures, primarily sexual selection and thermoregulation, have received considerable attention, while others, such as disease risk and parental effects, offer promising avenues to explore. As well as reviewing the existing evidence from both empirical studies and theoretical modelling, we highlight hypotheses that could benefit from further investigation in aposematic species. Finally by collating known instances of variation in warning signals, we provide a valuable resource for understanding the taxonomic spread of diversity in aposematic signalling and with which to direct future research. A greater appreciation of the extent of variation in aposematic species, and of the selective pressures and constraints which contribute to this once‐paradoxical phenomenon, yields a new perspective for the field of aposematic signalling.

## I. INTRODUCTION

Aposematic prey use warning signals to advertise their defences or unprofitability to potential predators (Poulton, 1890; Cott, 1940). Since Fritz Müller's (1879) first insights into the dynamics of aposematic species, selection from predators has generally been assumed to favour convergence in warning signals, as this decreases prey mortality during predator avoidance learning (Endler & Greenwood, 1988; Ruxton, Sherratt & Speed, 2004; Sherratt, 2008). Traditional theory holds that aposematic prey benefit from ‘strength in numbers’, as predators should learn an association between a signal and an aversive stimulus more rapidly and more effectively if they encounter it with greater frequency. Conversely, any aberrant forms of the warning signal, deviating from the ‘normative’ pattern (the average pattern or most common morph in the population) should increase mistaken attacks by predators, decreasing the effectiveness and speed of predator learning. Individuals with the ‘normative’ pattern thus benefit from the frequency of that phenotype and incur a reduced predation rate, whereas aberrant individuals do not have this benefit. Therefore, natural selection is thought to disfavour variation in aposematic patterns and favour monomorphism in warning signals (Poulton, 1890) – a hypothesis supported by many examples from the field (e.g. Mallet & Barton, 1989; Borer *et al.,* 2010; Chouteau, Arias & Joron, 2016). As a result, variation in aposematic signals has historically been considered paradoxical.

**Figure 1:**
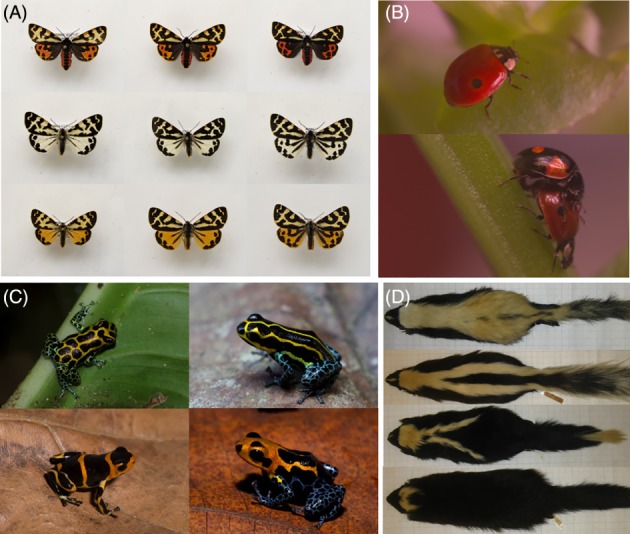
(A) White, yellow, and yellow/red morphs of the wood tiger moth (*Arctia plantaginis*) each vary in the extent of their melanisation. (B) The two‐spot ladybird (Adalia bipunctata) has numerous morphs including the typical melanic and non‐melanic forms shown here. (C) Morphs of the polytypic poison frog Ranitomeya imitator. (D) Continuous variation in stripe length and width in the North American striped skunk (Mephitis mephitis).

Nevertheless, variation in warning signals is found at several levels, from individual to population and species‐level differences, and recent research has led to a renewed interest in this diversity (Arenas & Stevens, 2017). The degree to which any one aposematic pattern enhances fitness is a product of many different selective pressures, ranging from predator–prey interactions and environmental conditions to trade‐offs with other signal functions (Ojala, Lindström & Mappes, 2007). In this review, we bring together some of the latest findings of experimental and theoretical work to address the role of these selection pressures, and help resolve the apparent paradox of variation in aposematic phenotypes. While aposematic signallers can utilise multiple modalities (e.g. visual displays, odours, sounds, behaviours), simultaneously or sequentially (Rowe & Halpin, 2013), visual signals have received the most attention, so we have focused our discussion on variation in colour and pattern in aposematic animals (see examples of aposematic variation in Fig. 1).

**Figure 2:**
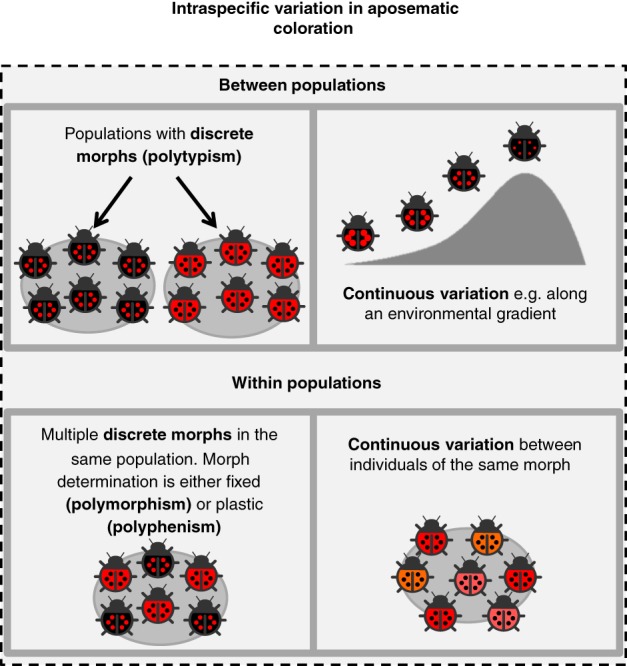
The levels of diversity in warning coloration discussed herein and associated terminology, with a hypothetical example using a single species of ladybird beetle.

Before addressing the processes underpinning variation in warning signals, it is necessary to understand the levels at which it occurs (Fig. 2). Warning coloration can vary allopatrically between different populations of the same species [e.g. polytypism (Mayr, 1963)] or sympatrically within populations [e.g. polymorphism (Ruxton *et al.,* 2004)]. The conspicuousness of signals, influenced by traits such as luminance and saturation, may also vary continuously among individuals of the same morph, temporally within a single individual across seasons or its life cycle, and among populations across a species' distribution range, forming a cline. Adding further complexity, more than one form of variation can occur simultaneously, and different components of the warning signal, such as size, pattern and hue, can vary independently, according to separate proximate mechanisms. Variation can be genetically determined (i.e. fixed), plastic, or shaped by the interaction of genes and the environment. The harlequin ladybird (*Harmonia axyridis*) for example, has multiple genetically determined morphs (Komai, 1956), but the extent of melanism within morphs has been shown to vary with developmental temperature (Knapp & Nedvěd, 2013). Considerable variation in aposematic signals is most difficult to explain at the intra‐population level, when alternative warning signal phenotypes co‐occur in single location (polymorphism, polyphenism, and continuous variation; Fig. 2). We have therefore focused our review on making sense of this poorly understood yet remarkably common phenomenon.

**Figure 3:**
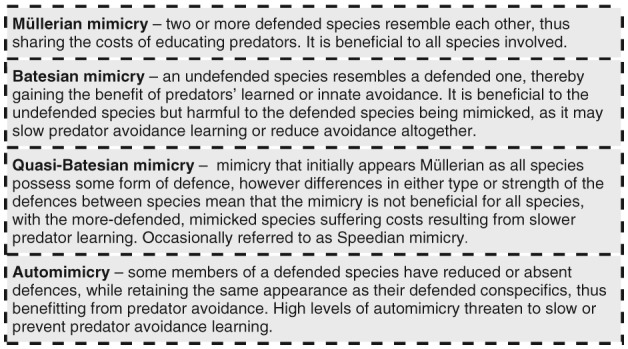
Definitions of the forms of mimicry discussed in this review.

Here we show how the complex biotic and abiotic environments in which species live give rise to a myriad of different selection pressures, which in turn lead to diversity in warning signals. This provides a general conceptual framework to explain when and why variation in aposematic patterns might exist. We begin by discussing the theory behind warning signal variation, then the demographic and genetic architecture that underpins it, before moving on to consider how variability in predation pressures can favour variation in warning signals, as opposed to monomorphy, even in mimicry systems (see Fig. 3 for mimicry). We then review how the multifunctionality of colour patterns can shape and favour diversity in aposematic signals. Finally, we summarise known cases of signal variation in aposematic species and discuss the taxonomic limitations of our current understanding of the diversity of warning signals. To showcase where and when warning signal variation occurs, and highlight possible systems in need of further study, we compiled a table of aposematic species in which variation has been described in the existing literature (see online Appendix S1 and Table S1). We find examples of warning signal variation in nearly every taxon in which we find aposematism (see online Table S1), suggesting that variation in warning signals is far more widespread than previously appreciated. Altogether, this review aims to demonstrate that variation in aposematic signalling should no longer be considered paradoxical, a new perspective that stands to advance our understanding of aposematic signalling.

## II. THEORY

Explaining the existence of phenotypic variation in the face of selection has long challenged evolutionary biologists and theoreticians (Bull, 1987; Roulin, 2004). The outstanding colour variation in aposematic species has been viewed as particularly problematic due to the pervasive view of predators as a ‘purifying’ selective pressure moving warning coloration towards monomorphism (Mallet & Joron, 1999). The majority of theoretical work investigating the factors that determine such colour variation focuses on Müllerian mimicry (Joron & Mallet, 1998; Sherratt, 2008), involving the evolution and maintenance of a shared warning signal in sympatric, aposematic species (Müller, 1879). While it may seem counterintuitive to discuss the theory behind the evolution of similarity to understand how variation might arise and be maintained, the factors responsible for creating or reducing variation in signal form are likely to be closely linked. That is, selection pressures for or against mimicry and within‐species ‘purifying’ selection may have many features in common.

Early models predicted that when there are multiple morphs present (whether they belong to one species or multiple species), an adaptive landscape characterized by multiple fitness peaks is generated, and predators should act to push the population as a whole to the highest adaptive peak by removing morphs defining lower adaptive peaks (generally the less common morphs), particularly when there are numerous prey types (e.g. Sherratt, 2002; Beatty, Beirinckx & Sherratt, 2004; Ruxton *et al.,* 2004). In a similar fashion, if variation within a population is not discrete, and the peaks are short with wide tails, then predators should push the population's adaptive peak up by removing outliers, i.e. those individuals most different from the ‘norm’ (Sherratt, 2006). Furthermore, where discrete variation occurs, the different phenotypes should evolve towards similarity as long as there is protective overlap between these distinct phenotypes in peak space, except when the phenotype is determined by a single locus (Turner, 1983). This occurs because overlapping space in the fitness landscape increases survival, and individuals that become increasingly more similar have overall higher survival (Mallet & Joron, 1999). This situation should only arise where there is a sufficient amount of overlap in fitness peaks in the adaptive landscape – if there is barely any overlap then the selection acting against phenotypes in the overlap area should be similar to that of a novel, unprotected form. In general, this scenario is more likely when there is one adaptive peak that is higher than others due to either population size or higher toxin load, in which case it should ‘capture’ the alternative species/morph (Turner, 1983).

These models predict that intraspecific warning signal variation would only persist under certain conditions. Firstly, variation can be maintained where population sizes are large (Plowright & Owen, 1980) and there is spatial or temporal variation in local predator communities combined with simple drift, resulting in a mosaic of different phenotypes (Franks & Noble, 2004; Ruxton *et al.,* 2004; Sherratt, 2006). Secondly, and slightly more contentiously, new morphs could arise and reach high local frequency through mechanisms such as bottlenecks, drift, mutation, *via* fluctuations in local ecological factors, or through relaxed selection due to a decline in predator abundance, causing peak shifts and the creation of new adaptive peaks (Turner & Mallet, 1996). Notably, the exact mechanisms by which this occurs are rarely covered in any greater detail than the above list, and are often treated as a ‘black box’. Herein, we attempt to flesh out both the mechanisms and circumstances that may lead to the creation and maintenance of these new phenotypes and corresponding adaptive peaks.

Once new peaks are created, theory posits that local predators should exert uniform, frequency‐dependent selection for all conspicuous species/morphs towards this new peak (Sheppard *et al.,* 1985). This stabilising selection can then work on surrounding populations via movement of hybrid clines or individuals migrating into new populations. This idea is known as shifting balance, and has been implicated in the evolution of geographical mosaic patterns in aposematic species and mimicry rings (Brown, Sheppard & Turner, 1974; Turner, 1983; Mallet, 2010; Chouteau & Angers, 2012). A key prediction of the shifting balance idea is that any form of polymorphism should be strongly selected against, and therefore temporary. Similarly, continuous variation in the aposematic signal should be generally selected against as stabilising selection should remove the most‐different individuals (i.e. those furthest from the ‘average’ appearance). This, of course, depends on predators being able to discriminate against and remember subtle differences in aposematic signal over time (see Section IV and Sherratt & Peet‐Paré, 2017).

Unfortunately, very little of the warning colour variation observed in wild populations meets the conditions outlined above. For example, multiple morphs of the same species are frequently found existing in the same locality (e.g. Brown & Benson, 1974; Borer *et al.,* 2010), often at low densities and/or low frequencies within a population (Chouteau *et al.,* 2017). Furthermore, the idea that such polymorphisms are likely to be transient and unstable has also been empirically challenged; for example, polymorphism in the poison frog *Oophaga pumilio* has been persistent on Bastimentos Island in Panama (Richards‐Zawacki, Yeager & Bart, 2013) and relaxed selection resulting from a decrease in predators produces a vastly reduced predation rate even on novel or intermediate forms (Chouteau & Angers, 2012). The mismatch between theory and empirical examples is in part due to the overly simplistic assumptions made about predator behaviour in earlier models. It is increasingly apparent that predator behaviour is more complex than early evolutionary models of warning coloration and mimicry allowed (Sherratt, 2008; Skelhorn, Halpin & Rowe, 2016), such as the early (and incorrect) assumption that predators sample a fixed number of prey to learn the association between signals and unprofitability (Rowland *et al.,* 2010a). The incorporation of some of this complexity in predator behaviour into models, e.g. optimal predator sampling strategies based on exploration–exploitation trade‐offs (Sherratt, 2011), has started to close the gap between theory and empirical examples resulting in scenarios where warning colour variation is predicted to arise within and among species (Aubier & Sherratt, 2015; Kikuchi & Sherratt, 2015).

As these more recent theoretical models demonstrate, less‐paradoxical predictions about the emergence and maintenance of multiple fitness peaks in warningly coloured species can be generated by incorporating predictions derived from empirical work on the complexity of predator behaviour. However, there is still a great deal of nuance in predator behaviour that has yet to be captured in theoretical models (see Section IV). It is also important to note that genetic mechanisms may facilitate or constrain variation (McLean & Stuart‐Fox, 2014; see Section III) and that independent fitness peaks can easily be reinforced by alternative biotic and abiotic selection pressures (other than predation) that may also act upon warning coloration (Calsbeek, Hasselquist & Clobert, 2010; see Section V). Below we outline these and other factors that future models could take into consideration, hopefully facilitating convergence of model predictions with the variation observable in the warning coloration of aposematic species.

## III. EVOLUTIONARY AND GENETIC CONSTRAINTS ON WARNING‐COLOUR DIVERSITY

Studies of the proximate mechanisms underlying aposematic variation have a limited taxonomic scope (but see Section VI and see online Table S1 for more possibilities), focusing primarily on *Heliconius* butterflies. Thus our review of the genetic and developmental pathways engendering diversity in warning colours is similarly largely based on insights gained from *Heliconius*.

### (1) Geographic isolation and range shifts

Many of the well‐studied polymorphic/polytypic aposematic species occur in the Neotropics, and consequently early hypotheses explaining polymorphisms and polytypisms relied on the Pleistocene refugium theory (Turner, 1965; Brown, 1979). This theory states that high rates of allopatric speciation/subspeciation resulted from fragmentation of tropical forests during climate warming, and then when climate cooled, and forests became continuous, species became sympatric (for discussion see Merrill *et al.,* 2015). The Pleistocene refugium theory has been invoked to explain the diversity of warning colours observed in poison frogs, neotropical Lepidoptera, and other tropical species, with refugia in Europe potentially playing a similar role for temperate species. However, this theory has recently been criticised and, in the case of *Heliconius*, time‐calibrated phylogenies indicate that diversity was present before the Pleistocene (Nelson *et al.,* 1990; Whinnett *et al.,* 2005; Dasmahapatra *et al.,* 2010; Kozak *et al.,* 2015; Merrill *et al.,* 2015). The current working hypothesis for how geographic or microhabitat variation has led to polymorphisms includes several stages. First, polytypisms arise through parapatric populations (populations with a narrow contact zone and low levels of gene flow) *via* a variety of non‐climatic mechanisms, such as genetic drift or adaptation to the local abiotic environment (Mallet, Jiggins & McMillan, 1998). Then, once populations are established, either gene flow continues or they eventually become sympatric, producing polymorphisms that may be transient (Mallet *et al.,* 1998; Joron & Iwasa, 2005). Polymorphisms/polytypisms can similarly arise due to earlier divergence of one clade, followed by subsequent mimicry by another clade (e.g. Symula, Schulte & Summers, 2001, 2003; Sanders, Malhotra & Thorpe, 2006).

### (2) Genetic basis of warning coloration

Investigations into both *Heliconius* and *Papilio* (swallowtail butterflies) species have shown that a handful of specific genetic loci and associated regulatory elements are responsible for the varied phenotypes these genera present (Kunte *et al.,* 2014; Kronforst & Papa, 2015; Nishikawa *et al.,* 2015). While a limited number of loci controlling colour and pattern would seem to be a fairly large constraint on the evolution of phenotypes, in both groups it is in fact the basis for extensive phenotypic diversity, resulting from repeated selection (Nadeau*,* 2016). For example, a number of key loci are known to control switches in pattern elements within the mimetic radiation of *Heliconius* butterflies [e.g. *WntA* (Martin *et al.,* 2012), *optix* (Reed *et al.,* 2011; Supple *et al.,* 2013) and *cortex* (Nadeau *et al.,* 2016)]. Kronforst & Papa (2015, p. 12) suggest that in *Heliconius* the phenotypic lability resulting from the influence of a small number of loci under strong selection creates a ‘virtually unlimited number of possible wing‐pattern phenotypes’. Intuitively, this makes sense as a smaller number of loci will increase each locus' contribution to the phenotype and thus each locus will be under stronger selection (Gavrilets & Vose, 2005). Ultimately a simplified genomic architecture facilitates the diversification of warning coloration.

Hybridisation and adaptive introgression among species have also contributed to the diversity of warning coloration in *Heliconius* (Mallet *et al.,* 1990; Gilbert*,* 2003; Heliconius Genome Consortium, 2012; Pardo‐Diaz *et al.,* 2012; Wallbank *et al.,* 2016). Although adaptive introgression and hybrid speciation both involve crossing individuals of different species, there is a difference that is worth noting as they are evolutionarily different mechanisms (Grant, Grant & Petren, 2005). Adaptive introgression results from gene flow from one species into the gene pool of another species through backcrossing of a hybrid with one of its parent species and can result in adaptive genes becoming incorporated back into the parental species (Grant *et al.,* 2005; Kronforst & Papa, 2015). Examples of adaptive introgression in natural systems are rare although reported cases do exist. Among *Heliconius* butterflies, *H. cydno* can hybridise with *H. melpomene*, and Pardo‐Diaz *et al*. (2012) found repeated introgression of adaptive alleles from *H. melpomene* in *H. timareta*. Hybrid speciation differs from adaptive introgression in that novel genomes are created from two parental species, which can lead to novel adaptive peaks in the landscape (Kronforst & Papa, 2015). Known hybrids include *H. heurrippa*, a hybrid of *H. melpomene* and *H. cydno* in the wild (Salazar *et al.,* 2005, 2008, 2010). Furthermore, *H. elevatus* was formed during a hybrid speciation event but is thought to have the colour patterns of *H. melpomene* introgressed into its genetic pool (Heliconius Genome Consortium, 2012), thus revealing a fine line between the dichotomy of introgression and hybridisation. There is also strong evidence that such hybrid‐trait speciation in *Heliconius* is promoted by tight genetic linkage between mate‐choice and colour‐pattern loci resulting in assortative mating based on wing colour patterns (Mavárez *et al.,* 2006; Kronforst, Kapan & Gilbert, 2006a; Melo *et al.,* 2009; Merrill *et al.,* 2011). For example, *H. cydno* and *H. pachinus* mate preference segregates with forewing colour in hybrids, indicating that colour preference and wing colour are controlled by loci that are pleiotropic effects of a single locus (Kronforst *et al.,* 2006b). Although our knowledge of hybrid speciation and adaptive introgression has come from Müllerian mimics*,* it is possible that non‐mimetic polymorphic aposematic coloration has resulted from both mechanisms.

Conversely, whilst some level of recombination can facilitate diversity in warning signals, too high a level has the potential to have a homogenising effect (Mayr, 1963), and hybridisation is not always adaptive (Arias *et al.,* 2016). In polymorphic populations, there should be tight linkage between loci to facilitate the coexistence of several combinations of congruous alleles, thus producing several different phenotypes (Merrill *et al.,* 2015). Genes that are closely linked (i.e. supergenes) facilitate multiple functional elements to segregate as a single Mendelian locus despite recombination elsewhere in the genome, and have been found to be associated with polymorphic mimicry (Brown & Benson, 1974; Charlesworth & Charlesworth, 1975; Turner, 1977b; Joron *et al.,* 2006; Thompson & Jiggins, 2014). *Heliconius numata* has several coexisting discrete mimetic phenotypes in the same population that are coded for by a single supergene (Joron *et al.,* 2011; Merrill *et al.,* 2015). Unsurprisingly, similar supergene architecture is not present in the sister species of *H. numata*, which do not have local polymorphisms (Huber *et al.,* 2015).

Many of the genes identified in *Heliconius* as controlling coloration are conserved across Lepidoptera (Nadeau, 2016; Nadeau *et al.,* 2016), which comprise a significant proportion of aposematic species and their mimics (see online Table S1). Whether similar genetic architecture underlies warning coloration polymorphisms in aposematic species outside this taxon is not yet clear. Work in ladybirds (Tan & Li, 1934; Komai, 1956; Majerus, 1994), colubrid snakes that are Batesian mimics (Davis Rabosky, Cox & Rabosky, 2016a), and a poison frog (Vestergaard *et al.,* 2015) indicate that morph variation in these species is also determined by a small number of gene loci. However, in contrast to the more complex supergene organisation seen in *H. numata*, mimetic warning coloration in colubrid snakes is the result of a much simpler multilocus system (Davis Rabosky *et al.,* 2016a). These differences can have important implications for evolutionary dynamics in mimicry, for example *via* their influence on evolutionary rate or even a subsequent evolutionary shift from warning coloration to crypsis, a phenomenon common in snakes but not in *Heliconius* (Davis Rabosky *et al.,* 2016b).

Given this evidence, it is clear that in order to understand how the genetic architecture of warning coloration enables or constrains morphological variation we need more information about the genes and gene networks at play, as well as a broader taxonomic coverage of the genetic architecture. Alongside the work already carried out on snakes, promising taxa include wasps (Perrard *et al.,* 2014) and ladybird beetles (Lee *et al.,* 2011). The latter are particularly intriguing as, unlike *Heliconius* spp., there is scant evidence of hybridisation, and for two highly polymorphic species (*H. axyridis* and *A. bipunctata*) multiple morphs have been produced in the laboratory that are scarce in the field (Majerus, 1994; Hodek, van Emden & Honek, 2012). Furthermore, recent work on the wood tiger moth *Arctia plantaginis* has revealed a negative genetic correlation between the efficacy of larval and adult warning coloration that likely contributes to the maintenance of observed variation in aposematic coloration at both life stages (Lindstedt *et al.,* 2016). Investigations into other such genetic correlations outside of *Heliconius*, for example between different components of the warning signals themselves (e.g. in *Pieris* butterflies; Kingsolver & Wiernasz, 1991), may therefore also prove fruitful to further our understanding of warning‐signal variation.

## IV. PREDATION AND SIGNAL VARIATION

Interactions between predators and defended prey lie at the heart of the paradox surrounding diversity in aposematism. While predation has traditionally been considered to favour monomorphy in warning signals, a growing appreciation of the differences in physiology, psychology and habitat use between predator species, populations, and individuals suggests that predator communities are in fact heterogeneous and dynamic selective agents. This generates diversity in predation risk and creates a significant opportunity for the maintenance of variation in aposematic prey.

### (1) Predators vary spatially, temporally, taxonomically, and individually

**Figure 4:**
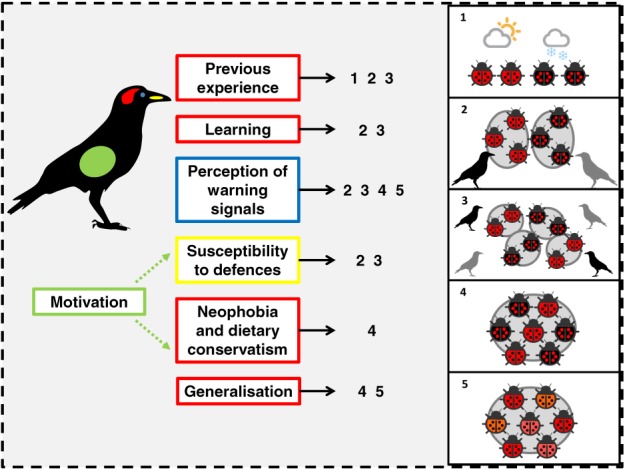
Types of variation in predators and the forms of warning‐signal variation they may promote: 1, temporal variation (for example seasonal polyphenism); 2, polytypism; 3, polymorphism within a metapopulation; 4, polymorphism; 5, continuous variation.

A predator's response to warningly coloured prey depends on both the prey's relative unprofitability and the conspicuousness of their visual signals (Mappes, Marples & Endler, 2005), so aposematic prey must carefully balance their investment in these two strategic components (Speed & Ruxton, 2007). Yet predators are also highly variable in their response to both chemical defences and visual cues. Therefore, the most adaptive tactic for defended prey will largely depend on the specific predator community in their immediate environment. Variation among predators and predator guilds can occur at several levels: among species, spatially among populations, temporally across seasons or an individual's lifetime, and at a finer scale among individuals (whether based on a stable behavioural type/syndrome or variable factors such as motivation), creating a mosaic of different selective pressures. In the following section, we suggest how variation in multiple predator traits, at different spatial and temporal scales, can facilitate the maintenance of different patterns of variation in prey signals (summarised in Fig. 4).

#### (a) *Types of variation in predators, and potential consequences*

For a given predator (species or individual), defended prey vary in their degree of unprofitability (Brower *et al.,* 1968), from mere distastefulness to deadly toxin loads. The impact of this difference is in part dependent on the specific predator and thus will differ among predators according to their susceptibility to specific toxins (Endler & Mappes, 2004; Mappes *et al.,* 2005), while the willingness of any individual to attack and consume defended prey will further be modulated by other factors, such as motivation and experience. Specialist predators, such as grosbeaks and orioles feeding on defended monarch butterflies, *Danaus plexippus* (Fink & Brower, 1981; Brower, 1988) or raptors preying on vipers (*Vipera* spp.; Valkonen *et al.,* 2012), can overcome the defences of aposematic animals, whether through resistance to their defences or careful handling. As such, attracting their attention with bright aposematic signals would be detrimental to prey survival. Tolerance of prey defences can vary across species but also among populations of predators; for example, some populations of garter snakes, *Thamnophis sirtalis*, have evolved resistance to newt tetrodotoxin (Geffeney, 2002). This may lead to polytypic or polymorphic variation in the conspicuousness of defended prey, following the distribution of more‐ or less‐tolerant predators across populations and microhabitats.

Predator sensory systems, including their perception of visual cues and other cognitive functions (e.g. ability to learn, remember and generalise between signals), may also facilitate the maintenance of polytypic and polymorphic variation among aposematic prey. The key sensory systems used for hunting differ among predator taxa, so, for the same defensive effect, prey may need to employ a diversity of signal forms to maximise their ‘avoid me’ signal efficacy (Guilford & Dawkins, 1991). Predation experiments with artificial prey demonstrate that only some predators respond to visual cues; for example, while avian predators avoid warningly coloured dendrobatid frog models, crabs and lizards do not (Willink *et al.,* 2014). Variation in the effectiveness of warning coloration when confronted with different predator communities may lead to conflicting selective pressures on prey signals. In Japan, the relative abundance of avian predators, which rely on vision when hunting, compared to mammalian predators, for whom visual properties are less relevant, may be responsible for the variation in the extent of red coloration in *Cynops pyrrhogaster* newts between island and mainland populations (Mochida, 2011). Among visually oriented predators themselves, there is considerable variation in perceptual abilities (Osorio & Vorobyev, 2008), suggesting that some predators could perceive or distinguish visual signals that others may not. In addition, sensory processing in the brain plays a role in determining key features influencing the effectiveness of warning signals, such as detectability, discriminability and memorability (Guilford & Dawkins, 1991). Finally, environmental conditions also affect the visibility and effectiveness of warning colours, dependent on ambient light and the characteristics of natural backgrounds (Endler, 1990, 1993; Bond & Kamil, 2006; Rojas, Rautiala & Mappes, 2014b); so aposematism overall, or some specific colour morphs, may be more effective in particular habitats.

Beyond perception of the signals, higher‐level cognitive processes may also influence predator responses to prey signals, and thus ultimately impact the adaptive value of conspicuousness and warning coloration. Generalisation between visual signals, whether they cannot be perceptually distinguished or are grouped together by higher‐order cognitive processes, is especially interesting, as it would effectively allow different colour morphs to co‐occur with equal fitness (Amézquita *et al.,* 2013; Richards‐Zawacki *et al.,* 2013; Stuckert, Venegas & Summers, 2014b; Rönkä *et al.,* 2018). For example, tests with multiple passerine species suggest that they differ in their ability to generalise prior experience of red firebugs (*Pyrrhocoris apterus*) to yellow morphs of this species (Exnerová *et al.,* 2006). Although it would not itself select for variation, generalisation between morphs could facilitate the maintenance of different forms (which could provide other selective benefits; see alternative selection pressures in Section V) in populations where predators tend not to distinguish between morphs.

Even if predators classify signals as distinct, further differences in their response will arise due to variation in general neophobia, cautiousness when handling novel prey, and dietary conservatism. These effects can potentially facilitate the evolution of novel conspicuous morphs (Marples, Roper & Harper, 1998; Thomas *et al.,* 2003, 2004; Exnerová *et al.,* 2006); although experimental evidence suggests dietary conservatism may not be sufficient to counteract positive frequency‐dependent selection against novel morphs when these are rare and conspicuous (Marples & Mappes, 2010). In some cases, innate avoidance of specific patterns plays an important role, as demonstrated by the aversion of naive turquoise‐browed motmots (*Eumomota superciliosa*) and great kiskadees (*Pitangus sulphuratus*) to coral snake (*Micrurus* spp.) patterns (Smith, 1975, 1977). Strong innate responses may allow polymorphisms in warning signals to evolve if the predators avoid a broad class of visual signals, such as all ringed patterns in the case of coral snakes. Finally, variability in the learning abilities of predators will affect the benefit of aposematic signalling for defended prey (Endler & Mappes, 2004; Mappes *et al.,* 2005). Recent work on domestic chicks (*Gallus gallus domesticus*) showed variation in avoidance learning among different breeds of this species. Chickens bred for high productivity were initially less wary of aposematic prey, but also formed weaker associations between signals and defences over time than the other breeds of chicken, leading to differential prey survival in laboratory experiments (Rowland, Fulford & Ruxton, 2017). Predators in the wild may also differ in their learning abilities, leading to variation in predation risk for aposematic prey with different signals, and are also likely to differ from domestic chickens. Further research on learning in more relevant predators could alter our expectations of predator capabilities and responses to aposematic prey; for example, evidence that predators can rapidly memorise many different signal forms would challenge the assumption of strong selection for aposematic signal monomorphy.

Classic experiments on neophobia and dietary conservatism in passerine birds also reveal further intraspecific variation, which cannot be attributed to factors such as differences in territory, experience or sex (Marples *et al.,* 1998). These could be linked to personality, known to affect both initial reactions to aposematic prey and the learning process (Exnerová *et al.,* 2010), or individual condition. A predator's level of hunger and current condition will determine its motivation and willingness to attack and consume risky prey, including warningly coloured individuals, which will impact the relative benefit of aposematic displays. Rather than rejecting aposematic prey outright, predators consider all available prey types to make adaptive foraging decisions, based on the relative costs of ingesting toxins *versus* the nutritional gain from consuming the prey (Barnett *et al.,* 2012). Experiments with European starlings (*Sturnus vulgaris*) suggest they can distinguish not only undefended from toxic prey, but also different levels of chemical defences, *via* taste‐rejection (Skelhorn & Rowe, 2006, 2009), as well as gaining nutritional information about the prey (Skelhorn *et al.,* 2016). This allows them to make educated decisions while foraging, depending on their motivation to feed; accordingly, starlings are more willing to consume defended prey when their own reserves are experimentally reduced (Barnett, Bateson & Rowe, 2007), early‐life or current conditions are harsher (Chatelain, Halpin & Rowe, 2013; Bloxham *et al.,* 2014), or the prey have greater nutritional value relative to their toxicity (Halpin, Skelhorn & Rowe, 2014; Smith, Halpin & Rowe, 2016). While there is a growing body of evidence, primarily from laboratory experiments, suggesting that varying levels of motivation affect prey choice by predators, how this may impact the survival of aposematic prey and selection pressures on signal form in the wild is not yet clear. The physiological mechanisms and cognitive processes responsible for these adaptive decisions are still relatively poorly known, but there is scope for mediation of this toxicity–nutrition trade‐off to vary among species, populations and personalities (Skelhorn *et al.,* 2016). Exploring how different predators deal with the trade‐offs associated with foraging in a natural setting, such as balancing the time required to assess the profitability of warningly coloured prey accurately, while managing their own exposure to predators and efficient foraging, would be extremely valuable for obtaining a more well‐rounded picture of predation risk for aposematic prey.

Motivation is not the only highly variable trait affecting predator responses to aposematic prey. Prior experience is critical in determining whether a predator will choose to attack and consume a prey item. This can vary widely across species and populations of predators, as traits such as dietary specialisations (Exnerová *et al.,* 2003; Ihalainen *et al.,* 2012) and territoriality (Endler & Rojas, 2009) affect which prey assemblages a predator may experience. For example, omnivorous and more specialised passerine birds respond differently when presented with aposematic invertebrates (Exnerová *et al.,* 2003). Similarly, great tits (*Parus major*) from Finland are more reluctant to attack aposematic prey than great tits from Bohemia, possibly due to a reduced exposure to warningly coloured invertebrates, and a higher proportion of neophobic and migratory birds in the population (Exnerová *et al.,* 2015). On a finer scale, a predator's level of experience will depend on the number of encounters with defended prey, so may differ between age classes (Lindström, Alatalo & Mappes, 1999). Seasonal fluctuations in overall predator naivety may occur as young predators learn to forage for themselves and sample aposematic prey for the first time, thereby impacting the relative benefits of conspicuousness and crypsis for defended prey at different times of the year (Mappes *et al.,* 2014) and potentially favouring seasonal polyphenism, as seen in striated shieldbugs, *Graphosoma lineatum* (Tullberg *et al.,* 2008; Johansen *et al.,* 2010).

Finally, variation in predator traits interacts with other forms of variation in the whole community of organisms in a given habitat, such that the characteristics of this community, and the interactions between all its members, will ultimately shape the selective pressures acting on warning signal form. From the predators' perspective, the presence, abundance and nutritional value of alternative prey, as well as the effort required to locate them and the toxin load already ingested, all impact the net benefits of attack (Turner & Speed, 1999; Sherratt, 2003; Rowland *et al.,* 2010c; Carle & Rowe, 2014; Skelhorn *et al.,* 2016), and the strength of selection for convergence in prey signals (Fig. 4; Kokko, Mappes & Lindström, 2003; Lindström *et al.,* 2004). The diversity of prey coloration within populations is equally important, not only in shaping predator experience, but also because of the demands it places on predators' cognitive skills. Selective pressures for signal uniformity may be relaxed in more complex communities, as predator learning is limited by their ability to memorise multiple signals and their associated risks and benefits (Ihalainen *et al.,* 2012). In an even broader ecological context, the predation risk experienced by the predators of aposematic prey themselves may also contribute to their response to warning signals (Lima & Dill, 1990), due to variable costs of exposure to predators incurred by longer prey‐handling times, or increased searching behaviour to find alternative prey. As such, differences in both prey and predator communities among populations, as well as spatio‐temporal heterogeneity within populations, combine to produce variable selection pressures affecting warning signal form.

#### (b) *Predator response to variation in prey toxicity, and its implications for aposematic variation*

Just as variation in predator communities was originally underappreciated, the variability of secondary defences, particularly chemical defences, in natural populations has long been neglected (Speed *et al.,* 2012). At the extreme end of this spectrum is automimicry, a phenomenon whereby some individuals within a population of aposematic animals have either extremely low levels of toxins or none at all (Brower, Brower & Corvino, 1967; Ruxton *et al.,* 2004). This seems to occur primarily in species that acquire either toxins or toxin precursors from their diet. Automimicry poses a problem for defended individuals because, similar to Batesian mimicry, it degrades the efficiency of the aposematic signal and thus any given individual in the population is more likely to be attacked (Fig. 3). Further, automimicry poses a problem for predators that may also experience negative side effects, for example by unintentionally consuming toxic prey after previous experience with a palatable individual of the same species (Ruxton *et al.,* 2004). Nevertheless, models indicate that automimicry may persist when there are two discrete levels of defence within a population and low predation pressures (Broom, Speed & Ruxton, 2005), or when defence is a continuous trait (and especially when defence levels trade off with fecundity; Svennungsen & Holen, 2007). Additionally, evidence indicates that automimicry may in fact not affect overall predation rates in a population when automimics are below 25% of the population (Skelhorn & Rowe, 2007). With respect to this review, automimicry is of interest as a potential intermediate step towards polymorphism, if the population of automimics begins to diverge into two different aposematic strategies. For example, in insects, females could evolve a preference for different host plants to oviposit on, which produces differential toxicity in the population and potentially different peaks in the adaptive landscape. Broom *et al*. (2005) have shown this to be a stable strategy and it could function as an intermediate step towards polymorphism *via* ecological mechanisms. Although theory would predict that the phenotype in the lower adaptive peak should evolve towards similarity with the higher peaked phenotype (e.g. Turner, 1983), there are alternative mechanisms that may maintain this (see Section V). Over time, this behaviour could become canalised and correlate with the aposematic signal as well. How common this is, or whether it occurs at all, is unknown. Automimicry may also be capable of creating polymorphisms in situations in which toxicity and colour are linked *via* some environmental trait. A plausible mechanism would be something akin to the resource‐allocation theory that has been supported by work on ladybird beetles (Blount *et al.,* 2009, 2012; see Section V), wherein some individuals acquire a chemical defence and others do not.

Similar to automimicry within a species, mimetic species are often unequally protected. This brings about a scenario known as quasi‐Batesian mimicry, occasionally referred to as Speedian mimicry (Speed, 1990; Fig. 3). Although mimicry has often been described as a binary scenario, i.e. either Batesian or Müllerian, there is evidence that it may be better represented as a spectrum, much as visual strategies are now perceived as a continuum ranging from crypsis to aposematism. Mimicry appearing to be Müllerian in nature may in fact be detrimental to one species and lead to quasi‐Batesian mimicry if there is a difference in the level of defence between the two mimetic species (Speed, 1990). Crucially, it is as yet unclear whether differences in toxicity and associated unpalatability actually produce quasi‐Batesian systems, or if variation between mimetic species with differing levels of toxins is ecologically irrelevant and these species have functionally mutualistic relationships (e.g. Rowland *et al.,* 2007; Stuckert *et al.,* 2014a). Similar to Batesian mimicry, local polymorphism may be beneficial to individuals of species with a low level of defence; if they can mimic different established aposematic species, they would gain a greater survival advantage, as the costs of mimicry would be spread across several model species (Speed, 1993; Ruxton *et al.,* 2004). Quasi‐Batesian mimicry may also put selective pressure on the less‐defended species to be more similar to the phenotype of the better‐defended species. This in turn may be sufficiently detrimental to the better‐defended species that they may experience selection away from the shared form (similar to Batesian mimicry). This could, theoretically, lead to an evolutionary chase between the model and the quasi‐Batesian mimics in a red queen chase scenario (Van Valen, 1973), particularly if selective pressures promote similar rates of adaptation in the two species. Furthermore, as discussed above, predators can make decisions based on both their nutritional level and toxin load, and therefore the availability of alternative, palatable prey may strongly influence the relationship between mimetic species, particularly if they differ in toxicity (Rowland *et al.,* 2010b). Including information on predator state in models of mimicry can lead to surprising outcomes; for example, two species that are visually distinct may both still benefit from the other species' presence even when toxins are not costly for predators to detoxify (Halpin, Skelhorn & Rowe, 2012; Halpin *et al.,* 2017). Additionally, differences in chemical defences (i.e. Batesian or quasi‐Batesian mimicry) could cause populations of a defended species to experience different coevolutionary trajectories (Laine, 2009), particularly when they are in geographic isolation. This could lead to polytypism, or polymorphism if the populations eventually become sympatric once more.

In reality, the role that variation in chemical defence has on populations and the evolution and maintenance of variation in colour phenotypes is largely speculative. This, in part, derives from a general uncertainty as to whether or not these differences in toxicity actually make ecological differences to predators. In general, we lack the empirical data to determine what this variation means to predators, or even why this variation occurs. This is a fairly substantial gap in our knowledge, one which could lead to a burgeoning subdiscipline.

### (2) Predator diversity contributes to the maintenance of variation in aposematic prey

#### (a) *The distribution of predator diversity shapes patterns of variation in prey*

Population‐level differences in predation regimes may facilitate the maintenance of continuous variation between populations of warningly coloured species, as seen in the red coloration of newts on Japanese islands (Mochida, 2011), or polytypisms. Within populations, many studies demonstrate greater predation risks for rare and novel conspicuous forms relative to locally abundant ones (Lindström *et al.,* 2001; Borer *et al.,* 2010), particularly in poison frogs (e.g. Noonan & Comeault, 2009) and *Heliconius* butterflies (e.g. Mallet & Barton, 1989; Chouteau *et al.,* 2016). These local predation pressures can produce a purifying selective force, driving populations towards distinct local phenotypes (Joron & Iwasa, 2005; Sherratt, 2006). In poison frogs, artificial predation experiments with models resembling distinct colour morphs of *Ranitomeya imitator* demonstrate that predation risk for these morphs varies geographically, favouring polytypisms (Chouteau & Angers, 2011).

On a smaller scale, differences between predator communities across microhabitats within a single population may facilitate the maintenance of polymorphisms in aposematic species and even contribute to speciation, as has been suggested for ithomiine butterflies (Mallet & Gilbert, 1995; Beccaloni, 1997; Elias *et al.,* 2008). In a recent study in Ecuador, butterflies with particular wing patterns were found at different frequencies among distinct microhabitats in the canopy (Willmott *et al.,* 2017). The community of avian predators likely to be encountered by these butterflies also covaried with these microhabitats, and artificial predation experiments suggested that predation risk experienced by specific wing patterns differed among microhabitats. Moreover, behavioural choices, such as temporal variation in activity or microhabitat selection, will enable aposematic prey to alter their conspicuousness and improve their chances of survival (Rojas, Devillechabrolle & Endler, 2014a; Arenas & Stevens, 2017), thus enabling multiple signal forms to coexist successfully.

#### (b) *Dealing with predator diversity within a population*

The presence of a diverse community of predators in a single location may favour variability in warning signals, so as to mitigate overall predation risk. Variation in the extent of conspicuousness may be employed as a compromise strategy, whereby signals of intermediate visibility, but still distinct and recognisable, may deter predators that heed the signal without attracting too much attention from others. For example, the polytypic poison frogs *Oophaga granulifera* and *O. pumilio* include morphs that are green and cryptic, others that are bright and truly ‘aposematic’, and intermediate phenotypes. This phenomenon seems to be related to behavioural phenotypes and attack rates by predators, as frogs from brighter populations are bolder and experience lower attack rates (Maan & Cummings, 2012; Willink *et al.,* 2013, 2014). Alternatively, a given signal may vary depending on the position of the observer. In distance‐dependent signalling, aposematic species possess pattern elements that make them appear cryptic from afar, yet conspicuous up close (Barnett & Cuthill, 2014; Barnett, Scott‐Samuel & Cuthill, 2016). Examples include *Vipera* snakes (Valkonen *et al.,* 2012), some butterfly larvae (Tullberg, Merilaita & Wiklund, 2005; Bohlin, Tullberg & Merilaita, 2008) and spotted skunks (*Spilogale* spp.), which are difficult to detect unless viewed closely (Caro *et al.,* 2013). Thus, prey coloration is not always exclusively cryptic or aposematic, but rather forms a continuum between camouflage and warning coloration, which can be manipulated to the prey's advantage.

Diversity within a population of predators can also maintain fixed variation within an aposematic prey population, under certain circumstances. Contrary to traditional theories of Müllerian mimicry, positive frequency‐dependent selection is not ubiquitous (Greenwood, Wood & Batchelor, 1981; Amézquita *et al.,* 2013; Richards‐Zawacki *et al.,* 2013). Müller's more simplistic assumptions about the relations between predators and prey, such as the fixed numbers of prey encounters required for learning, have since been replaced by a greater understanding of the complexity of predator communities. Considering the number of variables potentially affecting the overall outcome of foraging decisions by predators, a broad range of different selection regimes should be expected (Stevens & Ruxton, 2012; Aubier & Sherratt, 2015) including spatiotemporal variation in selection even within a single population of prey.

In particular, several processes may lead to negative frequency‐dependent selection, facilitating the maintenance of polymorphisms within populations (Svensson, Abbott & Härdling, 2005; Olendorf *et al.,* 2006). Foraging predators must constantly balance the costs and benefits of concentrating on prey they know to be profitable, or sampling unfamiliar prey items, which could be more valuable or potentially harmful. Optimal‐sampling theory predicts that these adaptive decisions will depend on the likelihood that a prey item is defended, and the probability that the predator will encounter this type of prey again. In the context of warning coloration, it suggests that rarer aposematic morphs should be avoided, as predators learn about profitability from their past experiences of more regularly encountered prey (Sherratt, 2011; Aubier & Sherratt, 2015). Search‐image formation, more‐efficient handling of commonly encountered prey, and the potential costs of gathering information about the profitability of unknown items will all encourage predation of common forms (Skelhorn *et al.,* 2016). Whether a predator will decide to attack common defended prey will also depend on prey toxicity within the community; for example, relatively weak defences or few palatable alternatives will favour predation on common aposematic forms, thus promoting polymorphism (Greenwood *et al.,* 1981).

The effect of predator community in different populations may even override expectations based on positive frequency‐dependent selection. Yellow and white morphs of male wood tiger moths occur at different frequencies across Europe, but local morph frequency does not always predict survival in artificial predation experiments. In one study, predation of the two morphs varied according to the community of bird species present, with yellow morphs being more successful in communities dominated by Paridae (tits, in Northern Europe), rather than Prunellidae, represented by the dunnock *Prunella modularis* (Nokelainen *et al.,* 2014). This suggests that understanding the characteristics of the relevant predator community may be the most important means of predicting signal evolution. In a general framework, modelling the evolution of a simple polymorphic prey population, with two morphs differing in conspicuousness and facing a mix of predators that differ in their tolerance of the prey defences, demonstrates several possible outcomes (Endler & Mappes, 2004). Depending on the proportion of predators choosing to avoid the prey, the population may become monomorphic for either the more or less visible morph, or, if both predator types occur in similar numbers, the polymorphism may be maintained. Experiments with firebugs and wild‐caught birds suggests that if a new colour morph of a defended species appears within a population, neophobia alone is unlikely to overcome purifying selection and enable the persistence of the new form (Exnerová *et al.,* 2006). However, evolutionary modelling suggests that a combination of dietary wariness, interacting with overall predation risk and signal conspicuousness will favour diversity in warning signals within populations, with or without frequency‐dependent selection (Franks & Oxford, 2009). Moreover, the results of simulations based on selection regimes observed in polymorphic species such as *Cepaea* land snails, *Oophaga* poison frogs, *Sonora* snakes and *Heliconius* butterflies suggest that differences in the range of predators, operating in small local populations or across multiple populations at a regional scale, can promote a mosaic of polymorphisms in prey, without invoking any additional mechanisms favouring diversity (Holmes, Grundler & Davis Rabosky, 2017). Multiple ways in which predators and predator communities may differ can thus ultimately affect selective pressures leading to diversity in warning coloration.

## V. THE MULTIFUNCTIONALITY OF APOSEMATIC SIGNALS

While predation is – by definition – the selective pressure driving aposematism, warning coloration is also subject to many other, potentially antagonistic, factors. These can be abiotic or biotic, the latter including both intraspecific and interspecific interactions. Several, such as thermoregulation and sexual selection, are already well studied in the context of warning‐signal polymorphism and polytypism, while others, including parental and early‐life effects, have only recently been recognised as potential factors generating and maintaining variation in coloration. Such selection pressures may be complementary to predation, augmenting its effect on aposematic phenotype, or alternatively may oppose the effect of the selective pressure of predation, producing more than one phenotypic optimum and enabling signal variation. These conflicting selection pressures can influence the abundance of different, genetically determined, morphs among populations and within a population (polymorphism), specific morph expression (polyphenism), and also more continuous colour variation within morphs (e.g. variation in conspicuousness; Figs 1 and 2).

### (1) Abiotic selection pressures

#### (a) *Temperature and melanism*

One clear example of a trait that is important for multiple aspects of an organism's fitness is melanisation. Melanin creates the black patterns seen in many of the classic aposematic signals across multiple taxa, from insects to mammals, as well as underlying the structural, iridescent, colours recently shown to act as aposematic signals in many bugs and beetles (Fabricant *et al.,* 2013; Fabricant *et al.,* 2014). The pigment also increases an organism's ability to absorb radiation (Clusella‐Trullas, van Wyk & Spotila, 2007; Hetem *et al.,* 2009) providing fitness benefits for individuals in cooler environments through improved thermoregulation (de Jong, Gussekloo & Brakefield, 1996). However, while increased melanisation provides fitness benefits for aposematic species in some instances (Clusella‐Trullas *et al.,* 2007; Lindstedt, Lindström & Mappes, 2009b), it also has associated costs. Melanic pigmentation often forms a key part of aposematic coloration, yet recent evidence suggests the contrast between a signal and its background (dictated by the chromatic component of the signal) as opposed to internal contrast, is the more important determinant of aposematic signal detectability (Arenas, Troscianko & Stevens, 2014). This may help to explain the much higher level of predation risk associated with melanism in aposematic species (Hegna *et al.,* 2013; Arenas, Walter & Stevens, 2015).

The trade‐off between the positive/thermoregulatory benefits and negative/predation‐risk costs of melanisation are well explored in aposematic species in relation to temperature (e.g. *Arctia plantaginis*; Hegna *et al.,* 2013). Variation in temperature is known to contribute to within‐ morph plastic adjustment of the levels of melanism in warning signals, for example spot‐size in ladybirds (Michie *et al.,* 2010, 2011), contributing to continuous variation in signal expression within and among populations. Seasonal fluctuations in temperature and changes in predation (see Section IV) likely promote melanism‐based polymorphism within populations of aposematic species. Even when the predation costs associated with the pigment are high, asymmetrical mate preferences, such that more‐melanic individuals have higher mating success, may contribute to the persistence of melanic morphs within populations (Saino *et al.,* 2013; Culumber *et al.,* 2014; Mishra & Omkar, 2014). The relative abundance of these melanic morphs within a population also increases with the benefits of improved thermoregulation (i.e. decreasing temperature), leading to both altitudinal and latitudinal clines in morph abundance (Clusella‐Trullas *et al.,* 2007). For example, the proportion of melanic morphs in populations of the two‐spot ladybird (*Adalia bipunctata*) is greater in higher, and therefore colder, latitudes (Brakefield*,* 1984) and these clines in morph abundance have been shown to alter in response to climate change (de Jong & Brakefield, 1998).

Melanin also has benefits associated with ultraviolet (UV) protection (Ortonne, 2002), immunocompetence (Dubovskiy *et al.,* 2013), and desiccation (King & Sinclair, 2015) and its abundance is plastically adjusted in response to increases in these challenges (Wilson *et al.,* 2001; Abram *et al.,* 2015; Välimäki *et al.,* 2015). Variation in these factors may act, like temperature, to enable the persistence of variation in the abundance of melanic morphs across spatial (polytypism) and temporal (polymorphism) scales. The selection landscape determining both the abundance of melanic morphs within aposematic populations and the degree of melanism within morphs themselves will thus consist of multiple competing selection pressures, beyond simply temperature and predation risk.

#### (b) *Resource availability*

The production of warning signals requires adequate resources to deal with the associated cost of signal production, both the overall increase in energy expenditure and the associated oxidative stress burden (McGraw, 2005; Galván & Alonso‐Alvarez, 2008; Blount *et al.,* 2009), and in many cases specific access to dietary pigments such as carotenoids (Blount *et al.,* 2012). Experiments in red‐eyed tree frogs (*Agalychnis callidryas*), a non‐aposematic species, have shown that the amount of carotenoid pigments available at critical times during development influences the redness of their dorsum (Ogilvy, Preziosi & Fidgett, 2012). Signal expression in aposematic species is therefore likely to be strongly influenced by the availability and quality of food, particularly during early development (Monaghan, 2008; Blount *et al.,* 2009). Research indicates that this most commonly occurs in terms of variation in morph conspicuousness (i.e. saturation and luminance; Blount *et al.,* 2012) as opposed to the dietary determination of differently coloured morphs observed in the camouflaged caterpillars of numerous lepidoptera species (Greene, 1989; Fink, 1995). High‐quality diets during development can lead to the production of larger, brighter, and more colourful warning signals compared to low‐quality diets (Grill & Moore, 1998; Ojala *et al.,* 2007; Lindstedt *et al.,* 2009a). The way that individual foraging areas and populations map onto the landscape of differing resource availability is likely to determine the scale at which the consequent variation in conspicuousness occurs, such as among individuals (continuous within‐morph variation) or among populations, for example along a resource gradient (polytypism). Furthermore, early‐life diet does not always affect warning coloration (Grill & Moore, 1998; Flores *et al.,* 2013), the relationship between the two is likely complicated by the fact that warning coloration advertises an associated defence (Poulton, 1890; Summers *et al.,* 2015).

#### (c) *The resource‐allocation hypothesis and quantitative honesty*

The nature of the relationship between an aposematic signal and defence is complex and likely to play a role in the way resource availability shapes aposematic signal variation, especially within‐morph variation in conspicuousness. While aposematic species are inherently qualitatively honest, they may not necessarily be quantitatively honest (i.e. show a positive relationship between the level of signal and the level of defence). For example, positive relationships between conspicuousness and toxicity have been identified in a number of species [e.g. ladybird beetles (Bezzerides *et al.,* 2007; Blount *et al.,* 2012; Arenas *et al.,* 2015) and paper wasps (Vidal‐Cordero *et al.,* 2012)]. However, the association is not universally positive, with negative correlations between levels of signal and defence identified across sexes or populations of the same species (Daly & Myers, 1967; Wang, 2011; Blount *et al.,* 2012). Furthermore, in some groups it seems that related species can reach approximately equal protection from predators with multiple different signal–toxin strategies (Darst, Cummings & Cannatella, 2006). A number of theories have been proposed to explain these differences (comprehensively reviewed by Summers *et al.,* 2015). Research on the availability of resources, those used for coloration and preventing autotoxicity (antioxidants), has provided a feasible mechanism: the ‘resource‐allocation hypothesis’ (Blount *et al.,* 2009). In this model, coloration and chemical defence both utilise antioxidants which are commonly acquired from the diet. Thus, individuals have to balance investments in the signal and defence, or deal with a trade‐off between investing in the signal or the defence (Blount *et al.,* 2009). The model predicts that when resources are low individuals will signal honestly, whereas under high resource conditions quantitative honesty would degrade as individuals would preferentially invest in defence over warning coloration. These predictions indicate that the influence of spatial or temporal variation in resource availability upon warning‐signal conspicuousness is unlikely to be consistently linear.

Empirical tests of the resource‐allocation theory are scarce however, and results equivocal in their support, showing that resource variation can lead to both positive and negative relationships between signal and defence (Blount *et al.,* 2012). The predictions of theoretical models investigating how individuals might invest in each component of an aposematic signal when resources vary also differ, depending on whether the model assumes that individual conspicuousness is an intrinsic component of the defensive signal or can act as a stand‐alone defensive trait (Blount *et al.,* 2009; Holen & Svennungsen, 2012; Summers *et al.,* 2015). The latter refers to a scenario where the warning coloration alone elicits wariness or acts as a deterrent against predators through its conspicuousness or novelty (Guilford, 1994). Alternatively, other work has suggested that the honesty of aposematic signals is not mediated by the cost of production, but instead by costs imposed by predators, because predators are able to determine levels of protection rapidly while sampling potential prey (Guilford & Dawkins, 1995; Hurd & Enquist, 2005). It is therefore clear that whether individuals respond to increased resource availability with a concomitant increase in warning‐signal conspicuousness will depend on the mechanisms of honesty enforcement at work. Further work to clarify the mechanisms determining the honesty of signalling in aposematic species (Summers *et al.,* 2015) will therefore aid predictions about how spatial and temporal variation in resources will influence within‐ and between‐population variation in conspicuousness.

### (2) Biotic selection pressures

#### (a) *Disease and parasite load*

As the influence of resource availability on warning signals demonstrates, animal coloration is strongly influenced by factors that affect an individual's condition (Griffith, Parker & Olson, 2006). Disease and parasite load both negatively influence condition and consequently can lead to trade‐offs between immune function and signal expression (McGraw & Hill, 2000). For example, increased parasite load leads to generally duller coloration in fish and birds of both sexes (Martínez‐Padilla *et al.,* 2011; Ciccotto, Dresser & Mendelson, 2014). Currently it is uncertain how such factors may influence aposematic signals specifically, but based on the shared physiological basis of aposematic and non‐aposematic coloration (e.g. sexual signals), it is possible that a similar ‘condition‐dependent’ relationship may occur (Blount *et al.,* 2009).

How the melanic component of aposematic coloration will be influenced by parasites and disease is unlikely to be clear cut. In common with coloured parts of warning signals, the production of melanin has various associated costs which may lead to trade‐offs between the production of melanin for pigmentation and immune responses (Guindre‐Parker & Love, 2014). Under such a scenario, a negative relationship between melanin pigmentation and disease or parasite load can occur (Cotter *et al.,* 2008; Gangoso *et al.,* 2011) and may result in polytypisms if these loads vary spatially. However the association between melanisation and resistance to pathogens is not straightforward; for example, in invertebrates, cuticle melanisation acts directly in the protection of individuals from pathogens (Dubovskiy *et al.,* 2013). Melanic pigmentation is also highly heritable in both invertebrates and vertebrates (Roff & Fairbairn, 2013; Roulin & Ducrest, 2013). It has been suggested that in many species the association between melanic coloration and a suite of disease‐resistance characteristics is a consequence of linkage disequilibrium and/or pleiotropy (Roulin, 2016). The result is differential life‐history strategies between more‐ and less‐pigmented individuals of the same species, the associated fitness of which is environmentally dependent (Emaresi *et al.,* 2014).

The preference of parasites, particularly ovipositing parasites such as parasitic wasps, for specific colour morphs and for within‐morph conspicuousness (or traits correlated with within‐morph conspicuousness) may act as alternative selection pressures on warning coloration. Parasites may prefer one colour morph over another, as is the case for the aphid parasitoid wasp *Aphidius ervi* which preferentially lays eggs in pea aphids, *Acyrthosiphon pisum*, of a colour morph not favoured by predators (Losey *et al.,* 1997). As discussed previously, coloration in aposematic species may be quantitatively linked to chemical defence (Summers *et al.,* 2015), levels of which have been linked to decreased (Weldon *et al.,* 2006) and increased (Zvereva & Kozlov, 2016) parasitism risk, the latter being especially prevalent for specialist parasites (Al Abassi *et al.,* 2001). Chemical defences have even been hypothesised to arise as a mechanism of preventing parasitism, with subsequent predator avoidance a secondary benefit (Weldon *et al.,* 2006). This relationship between colour and defence may be further complicated by the fact that some chemical defences can also have antimicrobial properties (Mina *et al.,* 2015). It is therefore conceivable that in areas with high risk of parasitism, colour morphs or levels of conspicuousness less attractive to parasitoids may be selected for, either through the parasites' direct response to colour or their response to levels of the strongly associated chemical defence. This may be especially important if infection dramatically reduces host survival (e.g. *Dinocampus coccinellae*; Maure *et al.,* 2014).

In summary, disease has the potential to cause continuous variation in the chromatic and achromatic parts of an aposematic signal due to current infection, plastic changes at the individual level where infection stimulates increase in melanisation, and local adaptation *via* correlated trait responses if coloration is linked to factors such as immunocompetence and if the level of infection risk varies spatially. Pathogens may also cause local extinctions, or repeated bottlenecks, which can disrupt purifying selection and maintain colour variation (Gordon, 2013; Idris & Hassan, 2013). Meanwhile, parasitism is likely to influence both morph abundance and within‐morph conspicuousness in populations of aposematic species. These areas are ripe for exploration, and have huge potential for contributing to the understanding of diversity in aposematic coloration and the life‐history trade‐offs involved in its determination.

#### (b) *Interspecific interactions*

Although predator–prey relationships dominate the study of interspecies interactions, other forms can and do occur. One such example is reproductive interference, i.e. sexual interactions between members of different species (Gröning & Hochkirch, 2008; Burdfield‐Steel & Shuker, 2011). Since this is, by definition, costly, mate discrimination and avoidance of reproductive interference could constrain warning signals, particularly in cases of mimicry, where effective mimicry could have consequences for mate discrimination (Estrada & Jiggins, 2008; but see Llaurens, Joron & Théry, 2014). Thus, the purifying selection on colour and pattern imposed by predators could be counteracted by the costs of sexual or territorial harassment by heterospecifics. While this phenomenon has not been investigated in aposematic species, such harassment has been suggested to play an important role in the maintenance of female colour polymorphisms in odonates (Fincke, 2004, and references therein). Because avoidance of conspecific harassment has been shown to influence female colour in Batesian mimics (Cook *et al.,* 1994) this phenomenon may be worthy of further research.

#### (c) *Intraspecific interactions*

##### (i) *Mate choice and parental effects*

Mate choice can act either to reinforce or to disrupt the selection imposed on warning coloration by predators. The interaction between warning signals and sexual selection can occur when aposematic traits play a function in mate choice and recognition, or when there is a trade‐off between traits used in mate acquisition and those involved in predator defence. As anti‐predator defence is a key survival trait, we would expect that natural and sexual selection would work in tandem, with better protected individuals also gaining advantages during mating and reproduction, thus enforcing purifying selection on warning coloration. However, when this is not the case sexual selection may act to counter the effect of selection imposed by predators, allowing for polymorphism and other forms of warning‐signal variation to arise (e.g. Cummings & Crothers, 2013).

Sexual selection could also lead to sex‐specific differences in warning coloration. For example, increased brightness in male poison frogs could be the result of female preference for brighter males (Maan & Cummings, 2009; but see Meuche *et al.,* 2013). Whether such selection would lead to true polymorphism in the eyes of predators depends on the strength of the respective pressures, as well as the sensitivity of the signalling system itself to evolutionary inputs. In the case of poison frogs, the colour cues selected for by females (i.e. brightness) may be different from those selected by predators (i.e. hue). Indeed there is evidence that *Heliconiu*s and *Melinaea* co‐mimics show increased interspecies variation in colour combinations that are less visible to their avian predators, allowing for ‘cryptic’ signalling of species identity (Llaurens *et al.,* 2014) and similar patterns may exist for within‐species signalling. Alternatively, if the visual conspicuousness of both sexes is already very high, any increases as a result of sexual selection may have no effect on predator learning (Maan & Cummings, 2009; Crothers & Cummings, 2013). Variation in the strength of female preferences among populations (e.g. Maan & Cummings, 2009) may create divergent evolutionary trajectories in different populations, causing polytypisms to arise, whilst assortative mating or local morph preference can enable their persistence and even lead to the exaggeration of morph differences. In such cases, warning signals may constitute so‐called ‘magic’ traits, as they are both subject to ecological pressures from predators and contribute to non‐random mating, as is the case for many *Heliconius* species (Merrill, Chia & Nadeau, 2014). Assortative mating by morph may also occur if individuals in a population have different anti‐predator strategies. For example, associating with a conspicuous individual may increase your own risk of attack if you are cryptic (Segami Marzal *et al.,* 2017).

In addition to assortative mating, disassortative mating, where individuals prefer to mate with colour morphs different from themselves, can also occur. For example, in the polymorphic *Heliconius numata* females show a strong aversion to mating with males of their own morph, preferring instead males of a different morph to themselves. Thus, while males of rare morphs may suffer increased predation risk, they will also have a higher mating success with females of the common morph (Chouteau *et al.,* 2017). This may be the result of heterozygote advantage, which has been suggested as a possible mechanism for the maintenance of many polymorphisms (Hedrick, 2012).

However, warning signals may not always be indicators of mate quality. Instead, there may be trade‐offs between traits related to predator defence and those that grant higher mating success. In addition, while selection on aposematic‐signal colour patterns is expected to be positively frequency dependent (see Section II), it could potentially be opposed if negative frequency‐dependent sexual selection also acts on the signals. Evidence for both phenomena has been found in the wood tiger moth. In this species white males have less‐effective warning signals (Nokelainen *et al.,* 2012) than the yellow morph they coexist with, but gain higher mating success in white‐biased populations, despite showing no advantage over yellow males in offspring hatching success (Gordon *et al.,* 2015). This could be due to differences in flying behaviour and mating effort (Rojas, Gordon & Mappes, 2015). Thus, frequency‐dependent selection could allow yellow and white males to co‐exist, as whites compensate for their higher predation rate through increased mating success. Sexual selection may therefore allow for the maintenance of polymorphism within populations, in particular if it leads to, or is a consequence of, a trade‐off between anti‐predator defence and mating success/fecundity.

While we naturally expect mate choice to influence offspring aposematic phenotype *via* genetic inheritance, transgenerational non‐genetic effects are also likely to play a role, especially in determining continuous within‐morph variation in warning coloration (Winters *et al.,* 2014). It is increasingly clear that offspring phenotype is influenced non‐genetically *via* maternal investment in response to a multitude of abiotic and biotic variables present in the offspring environment (Wolf & Wade, 2009; Day & Bonduriansky, 2011). Mothers can alter offspring phenotype in response to mate ‘quality’ (‘differential allocation’; Ratikainen & Kokko, 2010) and reliable cues of the offspring environment (‘anticipatory maternal effects’; Marshall & Uller, 2007). In terms of mate choice, as mentioned above, male warning coloration has been shown to be influential in female mate choice in aposematic species (Nokelainen *et al.,* 2012; Mishra & Omkar, 2014). Theoretical and empirical work suggest two likely responses, that females will either increase investment in response to the brightness or colour of male warning coloration (‘positive differential allocation’; Sheldon, 2000; Horváthová, Nakagawa & Uller, 2012) or decrease it (‘negative differential allocation’; Saino *et al.,* 2002; Bolund, Schielzeth & Forstmeier, 2009). Recent work provides the first evidence that such a phenomenon may take place, with female *Adalia bipunctata* laying brighter eggs when mated with brighter males (Paul *et al.,* 2018). This is important as offspring aposematic phenotype in early life can persist into adulthood (Winters *et al.,* 2014) and such differential allocation could therefore facilitate the perpetuation of variation in male warning coloration through subsequent generations. However, it is worth noting that work on differential allocation has also shown that a female's reproductive response to signals of male ‘quality’ varies with female age and phenotype (Sheppard *et al.,* 2013). Combined with other effects linked to maternal phenotype, condition, or maternal response to the offspring environment, this differential maternal investment in response to male phenotype is likely to lead to a mosaic of continuous colour variation within and among populations of aposematic species.

##### (ii) *Social signals and competition*

If warning signals can be used as signals during mate choice, and there is evidence that they can, they may also play a role in other forms of intraspecific interactions such as male–male competition, dominance and territorial disputes. While there are many studies that show the importance of visual signals in such interactions [see Shreeve, 1987, Setchell & Wickings, 2005, López‐Idiáquez *et al.,* 2016 for examples], few have considered aposematic species. One example comes from work on male–male competition in the frog *O. pumilio*; male brightness affected both their own behaviour and the behaviour of other males towards them. Brighter focal males were more likely to approach intruders to their territory, and brighter intruders elicited more calls and approaches than dull ones (Crothers, Gering & Cummings, 2011). This suggests that continuous variation in male brightness may be a conditional signal in this species, and that male–male aggression may play a role in its maintenance within populations.

Intraspecific warning signals may also occur when conspecifics can benefit from signalling their presence to others, and by heeding such signals. This may arise when competition between conspecifics is particularly costly. Possible examples include larval coloration signalling the presence of existing larvae on potential host plants to ovipositing females in order to reduce larval cannibalism in the pipevine swallowtail butterfly *Battus philenor* (Papaj & Newsom, 2005) and bright colours in male damselflies signalling their presence to other males in order to reduce male–male mating attempts during scramble competition (Sherratt & Forbes, 2001). While intriguing, honest communication of this sort seems unlikely to result in signal polymorphism, as it should also favour a single signal. If however, signals are dishonest, with signallers attempting to deceive the conspecific receivers, then there is the potential for red queen/chase away selection dynamics to unfold, similar to those that may occur during Batesian mimicry. Dishonest intrasexual signalling has been suggested as a potential reason for females displaying male‐like coloration, as this deceives mate‐searching males and reduces male harassment in butterflies (Cook *et al.,* 1994) and damselflies (Hammers & Van Gossum, 2008).

As well as increased competition for resources, high conspecific density is linked to increased detectability by predators (Riipi *et al.,* 2001). In aposematic species such aggregations actually augment signal strength and thus predator deterrence (Finkbeiner, Briscoe & Reed, 2012; Rowland, Ruxton & Skelhorn, 2013), changing the costs and benefits of large conspecific densities. This is reflected in the developmental ‘phase shift’ of the desert locust (*Schistocerca gregaria*). Coloration in these toxic locusts changes from a cryptic phenotype to an aposematic yellow and black when raised at high conspecific densities (Sword, 1999). This shift in anti‐predator strategy is a response to the increased likelihood of detection by predators when the cryptic locusts are in large aggregations. Predators learn to associate prey with toxicity more quickly when they are conspicuous rather than cryptic (Sword *et al.,* 2000) and the detection costs of warning coloration are diminished by gregariousness (Gamberale & Tullberg, 1998; Riipi *et al.,* 2001). Conspecifics can therefore influence not only within‐ but between‐morph variation in aposematic signalling.

#### (d) *Age‐structured populations and senescence*

Although not an alternative selection pressure *per se*, the conspicuousness of warning signals might also be expected to change over the lifetime of an individual, closely tracking major physiological changes resulting from processes such as reproduction and senescence (Booth, 1990b). For example, when warning coloration has a dual role as a predator deterrent and mate attractant, there might be an increase in signal strength during the mating season (Örnborg *et al.,* 2002; Pérez‐Rodríguez, 2008). Reproduction is also costly and adult condition is often poorer post‐, relative to pre‐breeding (Stearns, 1992; Monaghan, Metcalfe & Torres, 2009). Such a decrease in condition may potentially have consequences for the conspicuousness or ‘quality’ of an individual's aposematic signal, especially in longer‐lived species with multiple reproductive bouts (Velando, Drummond & Torres, 2010). Senescence, on the other hand, is strongly linked to a general decline in phenotype (Rose, 1991), and the strength of aposematic signals may become less effective with age in the same way as other colour signals, for example the blue feet of male blue‐footed boobies *Sula nebouxii* (Torres & Velando, 2007), the yellow bibs of the common yellowthroat *Geothlypis trichas* (Freeman‐Gallant *et al.,* 2011), and the wings of the orange sulphur butterfly *Colias eurytheme* (Kemp, 2006).

The effects of reproduction on coloration also vary according to an individual's age and sex, leading to a mosaic of colour expression within a population resulting from individual variation in sex, age class, and reproductive status (Evans, Gustafsson & Sheldon, 2011; Grunst, Rotenberry & Grunst, 2014). Furthermore, holometabolous insects such as Lepidoptera naturally show dramatic changes in form as well as coloration throughout their lifetime (Booth, 1990b), and more subtle changes in coloration are common in hemimetabolous insects, such as true bugs, between different nymphs and adult forms. Incorporate the aforementioned genetic correlations between warning signals at different life stages, or warning signals and other life‐history traits, and the picture becomes even more complex (Lindstedt *et al.,* 2016). An explicit test of the effects of reproduction and senescence on warning coloration variation and efficiency, also taking into consideration possible genetic correlations, is therefore needed. This would then enable us to ascertain whether similar patterns occur in populations of aposematic species and therefore if variation in age class within a population contributes to individual variation in conspicuousness.

It is clear therefore that there are myriad different selective pressures with the potential to influence the warning signals of aposematic species, some of which have already been empirically demonstrated to be important, and others worthy of further research. These sources of influence may act in ways that can be diametrically opposed to, or act synergistically with, predation pressure (Table 1). These ‘alternative’ selection pressures are most likely to produce and maintain warning signal diversity if they: (*i*) produce negative frequency‐dependent selection favouring rare morphs (for example those described in *Heliconius* by Chouteau *et al.,* 2017), or (*ii*) act in combination with heterogeneous selection imposed by predators (as described in Section IV) to produce different optimal phenotypes either within or among populations. In the latter case, the resulting selection landscape and associated phenotypic optima are also likely to vary over space and time, further slowing phenotypic convergence and allowing within‐population signal diversity to persist for longer. For example, temperatures will vary not only among habitats but also depending on climatic and seasonal timescales, and selection pressures associated with mate choice will vary in intensity throughout the year, especially in those species with distinct breeding seasons. It is vitally important that future work investigating the role of these selection pressures in producing warning‐signal variation incorporates their potential interaction, both with each other and with predators (e.g. temperature and either predator motivation or intraspecific aggression). It seems only rational to predict that such a movement away from the consideration of selection pressures on warning coloration in isolation is likely to result in a better understanding of the complex patterns of signal variation seen in nature.

**Table 1:** Summary of key factors facilitating the maintenance of different levels of variation within and among aposematic species.

Factor	Effect	Predicted form of signal variation
Variation among predators	(1) Broad‐scale differences in physiology (differences in sensory capacities, toxin tolerance and cognition) and behaviour among species and populations of predators	Polytypism; polymorphism if predators are structured across microhabitats; continuous variation; seasonal variation
	(2) Differences in predator experience among species, populations, and temporally within populations	Polytypism; polymorphism if predators are structured across microhabitats; seasonal variation
	(3) Small‐scale differences in physiology and behaviour among individuals, linked to motivation or individual experience	Would relax purifying selection, potentially allowing polymorphism or continuous variation
Temperature	Lower temperatures favour melanic components of warning signals, whereas predation selects against melanic morphs	Polytypism; polymorphism; continuous variation across populations along altitudinal or latitudinal gradients; continuous variation within populations (linked to microclimate during development); polyphenism/seasonal variation
UV damage	Increased UV risk favours melanic components of warning signals, whereas predation selects against melanic morphs	Polytypism; polymorphism; continuous variation across populations along altitudinal or latitudinal gradients; continuous variation within populations (linked to microclimate during development); polyphenism/seasonal variation
Desiccation	Increased desiccation risk favours melanic components of warning signals, whereas predation selects against melanic morphs	Polytypism; polymorphism; continuous variation across populations along altitudinal or latitudinal gradients; continuous variation within populations (linked to microclimate during development); polyphenism/seasonal variation
Resource availability	Availability of resources influences investment in warning coloration, often *via* effect on signalling honesty	Continuous variation within or among populations; polytypism
Disease and parasite load	(1) Effect of infection on individual condition	Continuous variation within or among populations
	(2) Stimulation of melanisation by infection or trade‐offs between use of melanin for pigmentation or infection resistance	Continuous variation within or among populations
	(3) Correlated trait responses if coloration is linked to factors such as immunocompetence or parasitism risk	Polytypism; polymorphism; polyphenism
	(4) Pathogen‐driven local extinctions, or repeated bottlenecks, which disrupt purifying selection and maintain colour variation	Polytypism; polymorphism
Intraspecific signalling	Warning colours may also serve as social cues, for example of quality or social status	Polymorphism; sexual dichromatism; continuous variation within populations
Density and aggregation	Density of aposematic species can alter selective landscapes, particularly the influence of frequency‐dependent selection imposed by predators. Aggregation of aposematic species can have a similar effect (and play into predator psychology to decrease the likelihood of an attack).	Polytypism; polymorphism; polyphenism

## VI. TAXONOMIC OCCURRENCE OF WARNING‐SIGNAL POLYMORPHISM

In our search for variation in aposematic signals, we found examples in nearly every taxon in which we find aposematism (see online Table S1), suggesting that variation in warning signals is far more widespread than previously appreciated. Despite this taxonomic diversity, a disproportionate amount of research effort has focused on a limited number of taxa, most notably Lepidoptera (especially the Neotropical *Heliconius*) and, to a lesser extent, the dendrobatid poison frogs. While this has enabled researchers to delve deeply into the various mechanisms producing patterns of variation within these species, it is unclear whether their findings generalise to other taxonomic groups.

Aposematic research has, for the past century, focused predominantly on terrestrial insects and their avian predators, possibly due to the tractability of these systems. However, examples of aposematic colour and pattern variation in other taxa such as birds (Dumbacher *et al.,* 1992, 2008) and mammals (Hunter, 2009; Stankowich, Caro & Cox, 2011; Caro *et al.,* 2013) have more recently been revealed. Warning‐signal variation in several marine species has also begun to be investigated (Hanlon & Messenger, 1998; Cortesi & Cheney, 2010; Winters *et al.,* 2017), although the aposematic function of conspicuous coloration in aquatic environments has been questioned. This is due to limited light availability, poorly known predator visual systems and predator–prey interactions, and the lack of known defence mechanisms for many brightly coloured organisms in the marine environment (Pawlik, 2012). It is probably in the non‐animal kingdoms where aposematism has received the least attention, despite reported examples in plants (e.g. Cahn & Harper, 1976; Karageorgou, Buschmann & Manetas, 2008; Lev‐Yadun, 2009) and even fungi (Sherratt, Wilkinson & Bain, 2005). There is therefore a need for more comparative studies on different taxa with robust phylogenies and detailed ecological information in order to address the ultimate causes of signal variation across taxa. Furthermore, utilising other systems parallel to the traditional terrestrial avian–insect interactions, for example, terrestrial plant–herbivore or marine food webs, may well provide new insights into the selective pressures and commonalities creating aposematic variation.

## VII. CONCLUSIONS

(1) While predator‐enforced selection on aposematic species appears to favour signal monomorphy in some cases, a growing appreciation of animal sensory systems and of the complexity of predator psychology in particular is challenging the concept of the predator community as a single invariant selective agent.

(2) Investigations of continuous variation or polymorphisms in aposematic species should first assess whether and to what extent the differences between individual signals actually impact predation risk. The perceptual abilities and responses of relevant predators, natural conditions and the microhabitat structure shaping encounter rates between predators and prey are especially important considerations.

(3) Equally as important, a variety of biotic and abiotic selection pressures experienced by aposematic species can contribute to warning signal variation within and among populations, and may potentially act antagonistically or synergistically with predator selection (summary in Table 1). Testing the relevance of visual signals to other behaviours, such as mate choice or thermoregulation, as informed by the natural history of the study species, will help piece together a more complex picture of the selective landscape driving signal variation.

(4) Moving forward, the field of aposematism should step away from the paradigm that warning signals are entirely determined by a uniform class of predators (generally birds), and instead consider both the strength of selection imposed by predators and alternative selective forces. Future work on aposematic species should adopt a more holistic approach to understanding colour and pattern, applying the tools of behavioural ecology, physiology and genetics to assess the relative power of predation *versus* other selective pressures in producing specific phenotypes.

(5) Broadening the taxonomic spread of research on warning signals and focusing on less well‐studied systems, encompassing different types of predators, would also help build a more comprehensive picture of the selective pressures determining variation in aposematism.

(6) Despite an overwhelmingly narrow research focus on predation pressures as the primary determinant of warning coloration, aposematism is affected by a range of forces, of which predation may not necessarily always be the most important. At the outset of this review we asked whether variation in warning coloration is a paradox or if it is the norm. It appears to be both; it is a paradox from the historical perspective that defines aposematic pressures *via* purifying selection enforced by predators, and the norm if we consider the empirical data and alternative selective pressures facing these species.

## VIII. ACKNOWLEDGEMENTS

We thank J. Mappes, K. Summers, M. Stevens, the organisers (Sensory Ecology Group) and attendees of the Anti‐Predator Coloration Symposium in Cornwall 2016 for discussions and inspiration. We would also like to thank K. Summers and J. Mappes for support and comments, as well as Mathieu Chouteau and an anonymous reviewer for their valuable input. Publication was funded by a BBSRC SWBio DTP studentship (award ref. 1355867). Data access statement: This is a review article and did not generate any new data; all data underlying this study is cited in the references.

## Supporting information


**Appendix S1.** Methods for compiling Table S1.

**Table S1.** Examples of warning‐colour variation described in existing literature.
