# Supplementary material for: Diversity in warning coloration: selective paradox or the norm?
Source: Biol Rev Camb Philos Soc. 2018 Aug 27;94(2):388–414. doi: 10.1111/brv.12460 (PMC6446817; doi:10.1111/brv.12460)
Supplement: Supplementary file 1 — Appendix S1. Methods for compiling Table S1. [file BRV-94-388-s001.docx]

**Appendix S1: Methods for compiling Table S1.**

Table S1 was assembled using both traditional literature searches and the authors’ own knowledge. In order to reduce the likelihood that any study systems were omitted, we then carried out a standardised literature search. The search terms used were all combinations (*N* = 21) of (Aposematism, Aposematic, Warning signal, Warning colour/color, Warning colouration/coloration) and (Diversity, Variation, Polymorphism). Searches were carried out in *Google Scholar* between May and June 2017 and the first 50 hits, sorted by relevance, were assessed. Any new species, or forms of variation in already included species, that were found were added to the table. Thus we are confident that Table S1 accurately represents the existing literature. As some species are represented by a single study or observation it was not always clear which forms of variation were occurring; in these cases suspected, but unproven, types of variation are denoted. Putatively aposematic cases were included when there was a clear indication of the taxa in question possessing defences against predation.

**Table S1.** (Provided as a separate file). Examples of warning colour variation described in existing literature. The types of warning colour variation reported for each taxon are denoted with crosses (x) in the table. Suspected, but unverified types of variation are denoted with question marks (?). In addition, a short written description of the type of colour variation, including within-population individual variation, is provided.
